# The association of neighbourhood and individual social capital with consistent self-rated health: a longitudinal study in Brazilian pregnant and postpartum women

**DOI:** 10.1186/1471-2393-13-1

**Published:** 2013-01-16

**Authors:** Gabriela A Lamarca, Maria do C Leal, Aubrey Sheiham, Mario V Vettore

**Affiliations:** 1 National School of Public Health, Oswaldo Cruz Foundation/FIOCRUZ, Rio de Janeiro, Brazil; 2 Department of Epidemiology and Public Health, University College London, London, UK; 3 Institute of Studies in Public Health, Federal University of Rio de Janeiro, Rio de Janeiro, Brazil

**Keywords:** Women’s health, Self-rated health, Dental public health, Social capital, Social network, Social support, multilevel analysis

## Abstract

**Background:**

Social conditions, social relationships and neighbourhood environment, the components of social capital, are important determinants of health. The objective of this study was to investigate the association of neighbourhood and individual social capital with consistent self-rated health in women between the first trimester of pregnancy and six months postpartum.

**Methods:**

A multilevel cohort study in 34 neighbourhoods was performed on 685 Brazilian women recruited at antenatal units in two cities in the State of Rio de Janeiro, Brazil. Self-rated health (SRH) was assessed in the 1st trimester of pregnancy (baseline) and six months after childbirth (follow-up). The participants were divided into two groups: 1. Good SRH – good SRH at baseline and follow-up, and, 2. Poor SRH – poor SRH at baseline and follow-up. Exploratory variables collected at baseline included neighbourhood social capital (neighbourhood-level variable), individual social capital (social support and social networks), demographic and socioeconomic characteristics, health-related behaviours and self-reported diseases. A hierarchical binomial multilevel analysis was performed to test the association between neighbourhood and individual social capital and SRH, adjusted for covariates.

**Results:**

The Good SRH group reported higher scores of social support and social networks than the Poor SRH group. Although low neighbourhood social capital was associated with poor SRH in crude analysis, the association was not significant when individual socio-demographic variables were included in the model. In the final model, women reporting poor SRH both at baseline and follow-up had lower levels of social support (positive social interaction) [OR 0.82 (95% CI: 0.73-0.90)] and a lower likelihood of friendship social networks [OR 0.61 (95% CI: 0.37-0.99)] than the Good SRH group. The characteristics that remained associated with poor SRH were low level of schooling, Black and Brown ethnicity, more children, urinary infection and water plumbing outside the house.

**Conclusions:**

Low individual social capital during pregnancy, considered here as social support and social network, was independently associated with poor SRH in women whereas neighbourhood social capital did not affect women’s SRH during pregnancy and the months thereafter. From pregnancy and up to six months postpartum, the effect of individual social capital explained better the consistency of SRH over time than neighbourhood social capital.

## Background

Social capital are features of social structure, such as interpersonal trust, networks and norms of mutual aid and reciprocity, which act as resources for individuals and facilitate cooperation and collective action [1]. In general, social capital can be conceptualized at both individual and contextual levels. Individual social capital is defined in terms of resources and support that are embedded within individual’s social network [2]. Contextual social capital emphasizes the resources that can be drawn upon by individuals to pursue collective aims by being interconnected. Contextual social capital is also referred to as collective social capital, and has been measured as neighbourhoods at country, state and local community levels [2]. Neighbourhood social capital is related to the relationships between social groups and their neighbourhoods and is largely based on day-to-day interaction between neighbours [1]. Neighbourhoods include social and environmental structures that not only include networks themselves, but also shared norms and mutual trust that facilitate cooperation for mutual benefit [3].

Cultural context and social rules influence reciprocity and mutual cooperation, and therefore affect the level of social capital. A report of the Brazilian Commission on Social Determinants of Health found that social trust is extremely poor among Brazilians [4]. Citizens feel that politicians in Brazil are not very trustworthy [5]. Trust in others is important to sustain collective feelings [6]. However, the low sense of effectiveness of politicians is reflected in the low level of social participation of younger Brazilians [6]. In Brazil, neighborhoods with low levels of social trust and low social control have high violence rates [7]. The increase in homicide rates has a strong social impact regarding neighbourhood security. Fear and insecurity are associated with feelings of impunity and indifference in the urban population as a result of violent crimes [7]. In addition, there is pessimism about safety and violence [8].

Social capital and its contextual dimensions, social trust, reciprocity, neighbourhood safety, neighbourhood support, social control, empowerment and political efficacy, has been associated with a large number of health outcomes; poor mental health [9,10], infant well-being [11], mortality [12,13], oral conditions [14-17], respiratory diseases [10], coronary diseases [18] and teenage pregnancy [19]. Social capital may play an important role in self-rated health (SRH) [20-24] as SRH is highly correlated with objective health measures [25,26]. Multilevel studies have shown an association between contextual social capital and SRH [27,28], although there is some inconsistency in the measurement of social capital. The studies reviewed by Engstöm *et al.*[28] reported that lack of contextual social capital (civic trust; political trust; civic participation; and/or political participation), was related to poor SRH in adults and young adolescents. Eriksson *et al.*[29], who investigated the associations between collective social capital and SRH in men and women from Sweden, showed that women living in very high social capital neighbourhoods were more likely to rate their health as good or fair compared to women living in neighbourhoods with very low social capital [29]. Different aspects of the neighbourhood environment are associated with people's SRH [20,21,23,30-33]. Neighbourhood safety and political participation, dimensions of neighbourhood social capital, are related to better SRH in women [31]. Furthermore, women living in states with low levels of women’s political participation were more likely to rate their health as poor [32].

The social environment related to neighbourhoods is particularly important for women's health [31,34,35], and has also been considered as a determinant of health during the gestational period [36,37]. Place of residence [38], neighbourhood poverty and deprivation [39-42], community participation [37] and neighbourhood crime rates [43] accounted for different rates of preterm birth, low birth weight, inappropriate weight gain [44], gestational hypertension [45] and miscarriage/perinatal death [39]. Moreover, social capital was associated with health-related behaviours during pregnancy [45] and the use of prenatal care [46]. However, studies on the influence of neighbourhood environment on pregnant and postpartum women’s health, including those focusing on social capital, concentrated on clinical outcomes of pregnancy [37,44,47]. No study has explored the association between neighbourhood-level social capital and subjective measures of health, such as self-rated health, in pregnant women despite of its relevance to both maternal and child health [28,48]. SRH has been used reliably in studies involving proximal causes of diseases in pregnant women and up to one year postpartum [28,48-50].

Although environmental characteristics embedded in the neighbourhood structure may be a potential determinant of subjective measures of health, social support and social networks, considered in this study as individual-level social capital [2,3,51,52], have been cited as risk factors for maternal well-being during pregnancy and postpartum [53-55]. Some observational and interventional studies showed that social support is linked to outcomes of pregnancy and health in the postpartum period [56]. Social support has been associated with low levels of stress after childbirth [57], and lack of social support was associated with poor SRH in the postpartum period [58,59]. Lack of social support and ongoing physical and emotional problems were more important for SRH than socio-demographic background [60]. Social support and social networks have been also associated with reproductive outcomes. Inadequate social networks and weak social support affected intrauterine growth, especially among socially deprived women [61].

Changes in women’s SRH during and after pregnancy are expected because of body changes, physical recovery, adaptation to motherhood and adverse factors related to the infant [60]. Existing medical conditions such as gestational hypertension and gestational diabetes may get worse during pregnancy. The most common adverse condition during pregnancy is urinary tract infections [62]. It causes more symptoms than other conditions and may lead to maternal and neonatal morbidity and mortality [63]. Diseases of the lower urinary tract occurring during pregnancy lead to more symptoms than other conditions. Pregnant women with the abovementioned diseases usually experience malaise, indisposition, discomfort, polyuria and lower abdominal pain [64], which may influences their SRH.

Despite the changes during pregnancy, SRH remained 'very good' or 'good' in the majority of pregnant women during one year postpartum [49]. However, the factors contributing to the consistency in SRH are unknown. Among the factors suggested, individual social capital, such as social networks that reflect the way women receive social support, is considered an important proximal determinant of their health [65]. It has been argued that different forms of individual social capital are protective resources that exert a buffering effect against life stresses and can improve emotional well-being of mothers [66], and therefore, may have a positive effect on their SRH.

**Figure 1:**
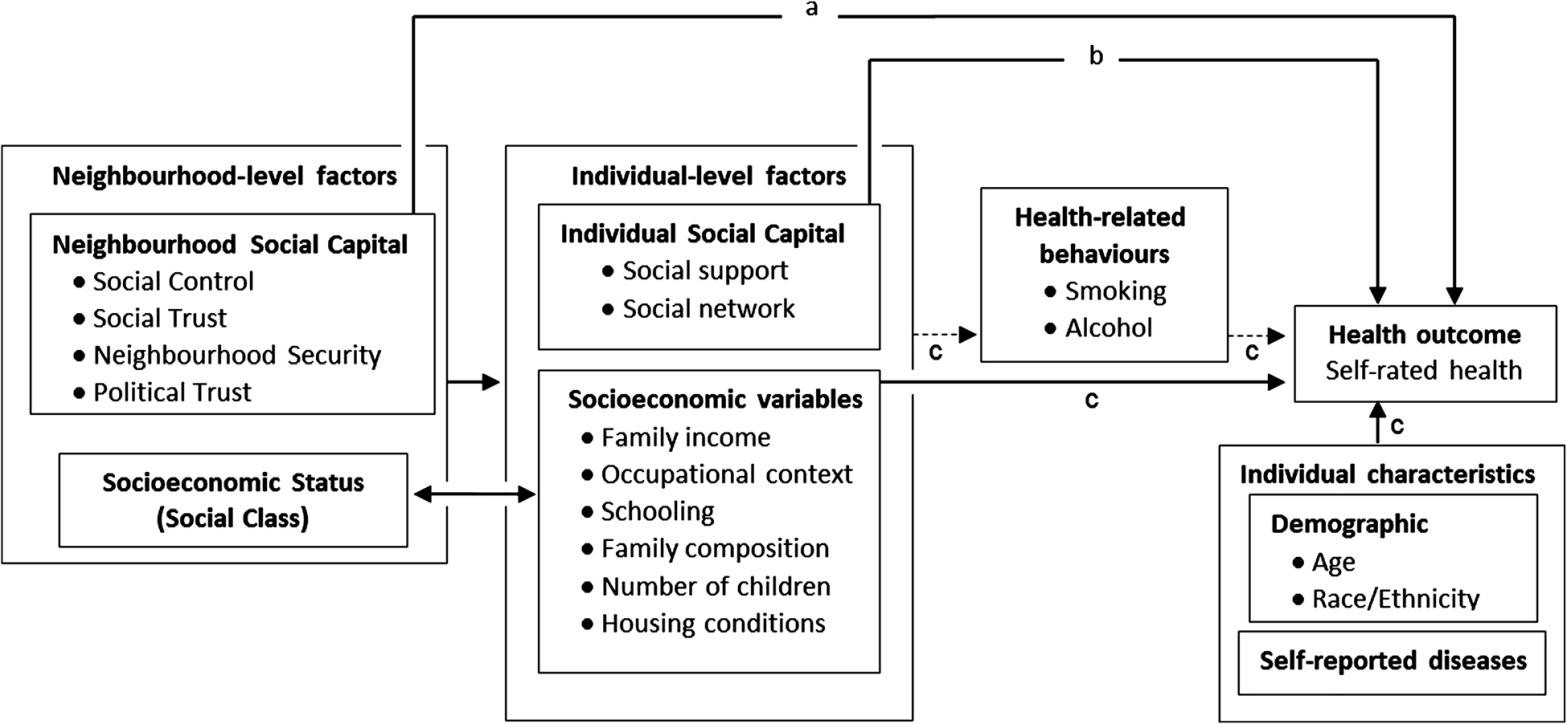
**Conceptual model of the research. **(**a**) Direct effect (**b**) Indirect or mediator effect (**c**) Confounders.

Despite the evidence showing a link between the place where women live and their health during pregnancy, the effect of neighbourhood-level social capital on SRH during pregnancy and for some time after childbirth is unknown and needs investigation. A multilevel longitudinal study was therefore conducted to gain a better understanding of what affects SRH in women during pregnancy and six months after delivery. It is important to distinguish the independent effects of contextual determinants (neighbourhood social capital) and individual determinants (social support and social networks) on SRH levels during pregnancy and during the postpartum period [49]. To address those research questions a theoretical framework encompassing a number of interlinked neighbourhood and individual-level characteristics was developed and employed in the present research (Figure 1). The objective of the study was to investigate the association of neighbourhood and individual social capital with self-rated health in women between the first trimester of pregnancy and six months postpartum.

## Methods

### Study site

This was a population-based cohort multilevel study conducted in two urban middle-sized cities in the State of Rio de Janeiro, Brazil. The main study, of which this is part, was designed to assess the relationship of social determinants with undesirable pregnancy outcomes, use of prenatal care [46], and child and mother’s health six months after childbirth. The two cities were selected because they had the following features: 1. considerable differences in *proxy* measures of social capital such as violence rates and per capita income: U$222 and U$101; 2. demographic similarities: population <300,000 inhabitants and birth rates between 130 and 170 births per 100,000 inhabitants [67,68]; and 3. coverage of antenatal care was above 90% in both cities and the provision of antenatal care was concentrated in few health care units facilitating recruitment of a representative sample of pregnant women in both cities.

### Settings

The participants were women who sought antenatal care at the public health care units administered by the National Health Care System ("Sistema Unico de Saude - SUS"). They were a representative sample of 95% of the women who were pregnant during the study period in both cities. In general, the use of prenatal care is almost universal in Brazil. Only 1.3% of live births did not benefit from prenatal care in Brazil from 1996 to 2006 [69]. Prenatal care commenced in the first trimester of pregnancy in 85.5% of pregnant women in the Southeast region of Brazil, where the study was conducted [69]. Approximately 15.0% of women were not included in the study because they did not seek antenatal care until after the first trimester. The prevalence of caesarean section in Brazil was 52.2% in 2010 [69], ranging from 58.2% (Southeast Region) to 41.7% (North Region) [68]. Caesarean section rates differ significantly between the private health sector (82%) and the public health sector (37%) [70,71]. The public sector deals with 75% of all deliveries in Brazil. In 2008, 3,861 women gave birth within the public health care system in City 1 of this study (1,603 – vaginal; 2,258 – caesarean) and 2,347 in City 2 (1,258 – vaginal; 1,087 – caesarean; 2 - not informed) [68].

### Test-retest study

Twenty interviewers were trained to conduct structured and standardized interviews. Then, a pilot study to test the understanding of questionnaires and a test-retest study were performed. The test-retest study was conducted to evaluate the reliability of the social capital questionnaires. Forty pregnant women were recruited at the same health care units of the main study. Intraclass Correlation Coefficient and Cronbach’s α were used to test reliability and internal consistency of the social support and social capital scales.

In the test-retest study, intraclass correlation coefficient of agreement findings for social capital questionnaire was 0.893 and ranged between 0.860 (emotional support) and 0.907 (material support) for the social support dimensions. Cronbach coefficient for social capital was 0.706 and ranged from 0.706 (affectionate support) to 0.863 (emotional support) for social support dimensions.

### Sample size calculation

The sample size calculation considered 25 as the average number of individuals per neighbourhood [72]. The prevalence of 20% of poor SRH [48] in low social capital areas and 5% in high social capital areas and sample intra-cluster coefficient of 0.017 were used in the calculation. The sample size was estimated to be 1125 pregnant women in 49 neighbourhoods with a significance level of 5% and power of 90%. The sample size was increased by 60% to allow for non-acceptances, losses to follow-up and changes in SRH. Therefore, 1750 pregnant women in 55 neighbourhoods were invited to participate.

### Study participants

The participants were women in the first trimester of pregnancy and living at their current address for at least 12 months and who did not change address between the different waves of the study. These criteria were used because neighbourhood social capital and individual social capital variables (social networks and social support) tend to be stable after some months living in the same place. First, the interviewers inspected the medical notes and chose pregnant women according to the selection criteria. All eligible pregnant women were invited to participate. They were informed about the objectives of the study. After obtaining their consent, the women were interviewed. Women who had a miscarriage or an abortion were excluded.

Primary data were collected through face-to-face individual interviews between October 2008 and December 2009. The baseline was conducted at the antenatal care units during the first trimester of pregnancy and the follow-up at 6 months postpartum (±180 days) at women’s houses.

Data regarding demographic and socioeconomic characteristics, health-related behaviours and individual social capital were collected in all interviews. Neighbourhood and individual social capital as well as individual socioeconomic and demographic characteristics were assessed at baseline. SRH was evaluated at baseline and follow-up. Different strategies were established to reduce the losses throughout follow-up.

**Figure 2:**
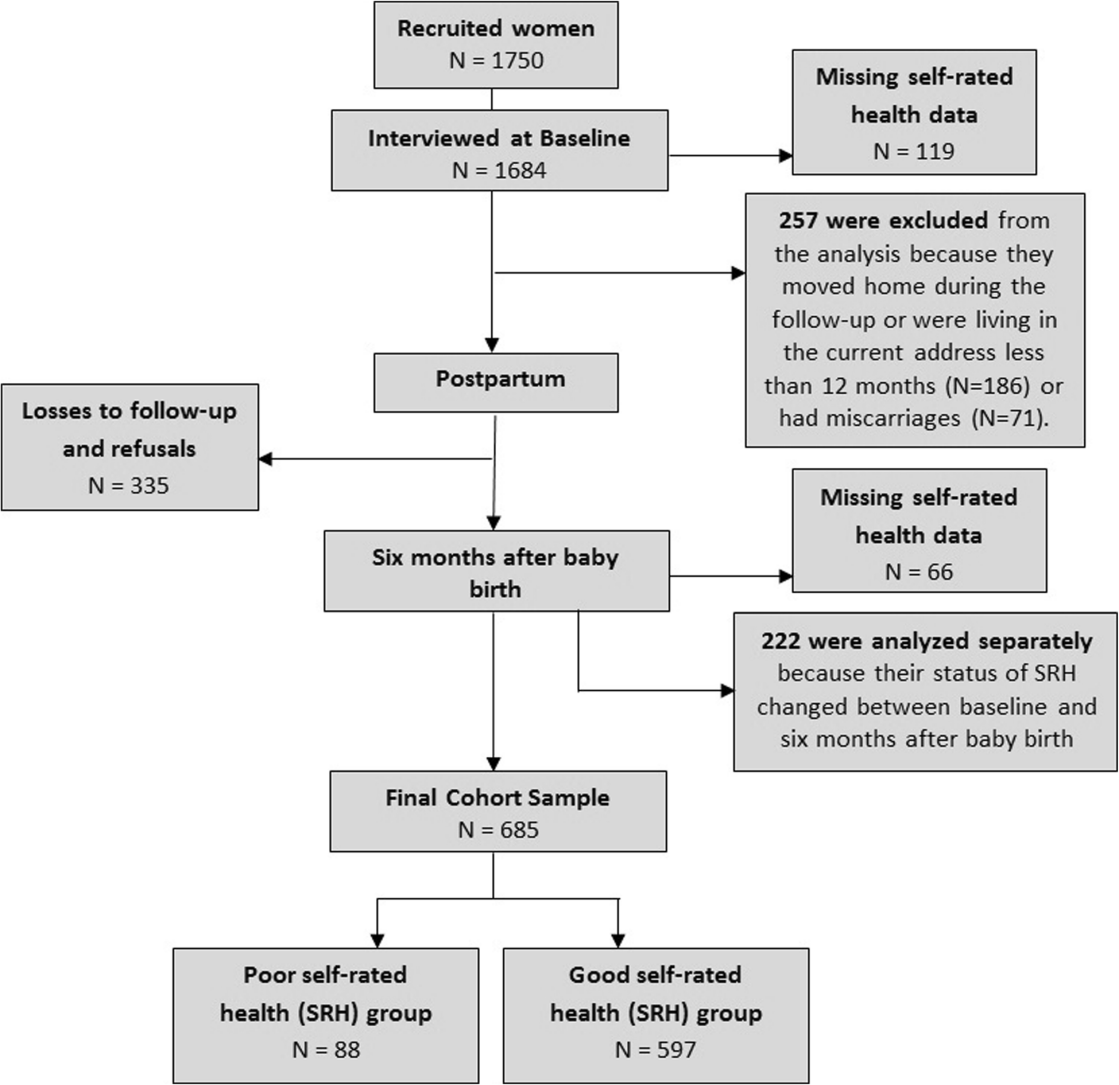
Flowchart of the sample.

Initially, 1750 pregnant women were invited to participate in the study. The acceptance rate was 96.2%. Of the 1684 women interviewed at baseline, 257 were excluded from the analysis because they moved home during the follow-up or were living in the current address for less than 12 months (N = 186) or had miscarriages (N = 71). The cumulative losses from baseline to follow-up were 19.9%, (335 women, including refusals). Missing data for SRH were 119 and 66 at baseline and follow-up, respectively. Of the 907 women with data on SRH, 222 were separately analyzed. Therefore, 685 women from 34 neighbourhoods composed the final sample. The sample included 597 (87.2%) women with good SRH and 88 with poor SRH (12.8%). That allowed detection of a 20% of difference in the prevalence of poor SRH between low and high social capital areas. The flowchart of the sample is presented in Figure 2.

### Exposures

#### Neighbourhood social capital

Although there are no generally agreed-upon standard criteria to measure neighbourhood social capital, four dimensions comprising social trust, social control, neighbourhood security and political efficacy have been confirmed by factorial analysis as relevant measures. Social trust refers to people's perception of trust, connectedness and solidarity in their neighbourhood [73]. Informal social control, defined as the willingness of neighbourhood residents to intervene in local problems, is an important mediator between neighbourhood structural conditions and crime [73]. Political efficacy refers to people’s perceptions of the political system and politicians [74]. Finally, because less violent communities are usually more equal and have more trust [75,76], the conceptual framework included people’s perception of security as a component of social capital, a dimension that has been used elsewhere [77]. To measure social trust and social control two core sets of questions were used from Sampson’s seminal paper on collective efficacy [73] and from Stafford *et al.*[78]. Items relating to political efficacy were from the American and British Political Action Surveys [74]. Neighbourhood security was assessed by items related to frequency of violent occurrences in the neighbourhood [73]. The social capital questionnaire is presented as Additional file 1.

The questions used to measure neighbourhood social capital were adapted from a study on adolescents in Brazil, which demonstrated adequate internal consistency for the 30 items of the scale (Cronbach’s alpha > 0.70) [14,15]. The empowerment dimension was not used because it loaded less than 0.30 in the factorial analysis in our sample [79]. Our questionnaire showed sufficient reliability and validity. Cronbach α coefficient for all scales was above 0.7 (a table of items and dimensions comprising the social capital index is available from the author). The neighbourhood social capital measure was created as follows: negative items were reverse-coded so that all items ranged from low to high social capital. Weighted and unweighted analysis produced similar results [15,16]. Since each subscale of the social capital questionnaire had different numbers of items, the final scores for each subscale were standardized from 0 to 100. In this way, the subscales were comparable and could be added up to form the neighbourhood-level social capital variable.

**Figure 3:**
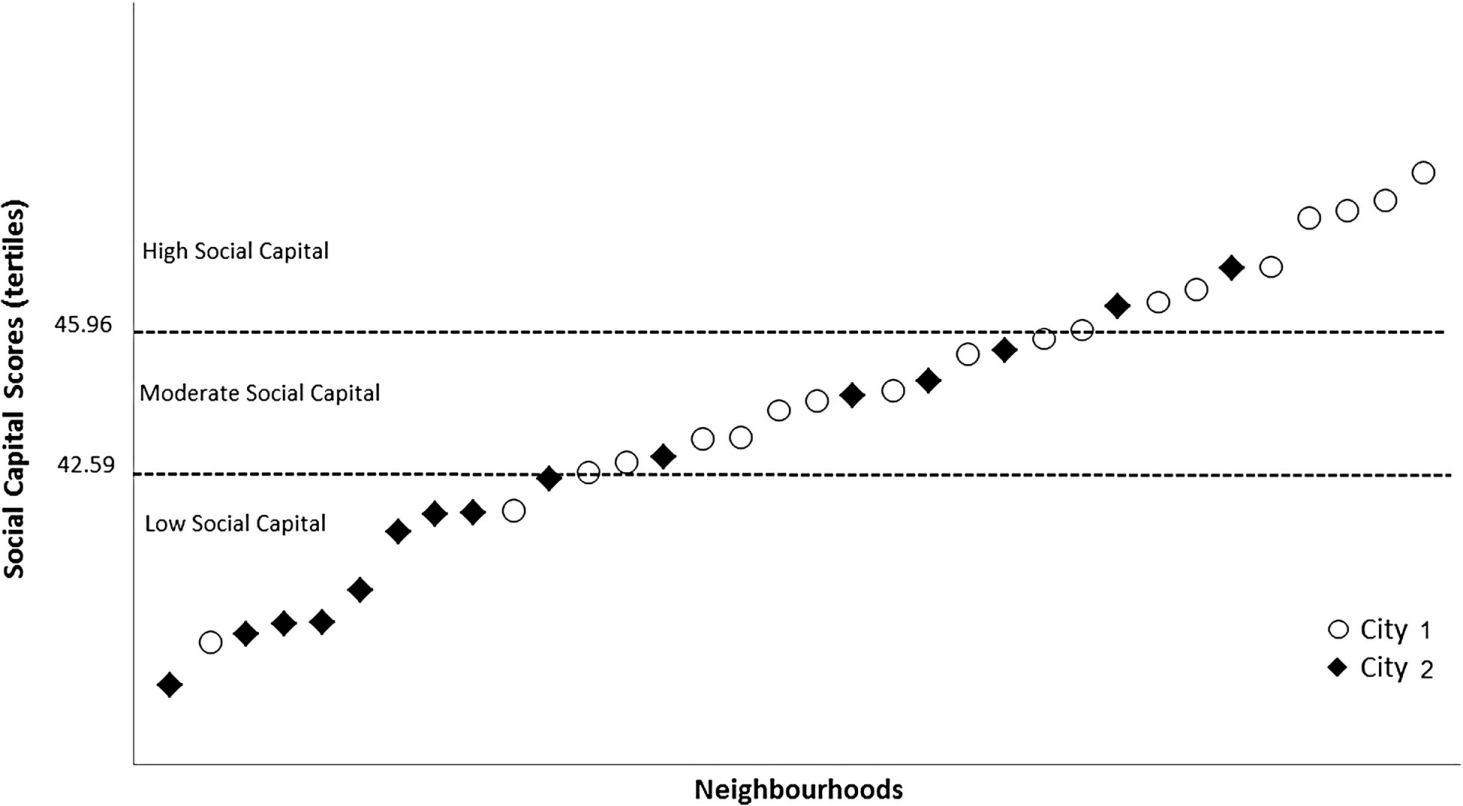
Distribution of the 34 neighbourhoods areas according to the tertiles of social capital scores: Low social capital neighbourhood area: score from 37.05 to 42.59; Moderate social capital neighbourhood area: score from 42.60 to 45.96; High social capital neighbourhood area: score from 45.97 to 50.51.

Items were chosen assuming they reflected the concept of social capital as neighbourhood characteristics. Therefore, questionnaire data on social capital were collected at individual level and then aggregated at area (neighbourhood) level according to address and residential zip code. The average number of respondents per neighbourhood was 20.2 women. The participants were grouped into 34 neighbourhood areas: 19 neighbourhoods in City 1 and 15 in City 2. The neighbourhoods were then categorized into three equal groups according to tertiles of the social capital score as follows: low (from 37.05 to 42.59), moderate (from 42.60 to 45.96), and high (from 45.97 to 50.51) neighbourhood social capital (Figure 3).

#### Individual social capital

Individual social capital was assessed by measuring social networks and social support. Social networks was considered as the "web" of social relationships surrounding the individual as well as their characteristics, or groups of people who have contact with, or with some form of social participation [80]. The questionnaire to assess social networks consisted of 5 questions on the person's relationships with family and friends, and their participation in social groups. Social networks questions are presented as Additional file 2.

Social support was considered as a system of formal and informal relationships through which individuals receive emotional support, material or information to cope with stressful emotional situations [81]. Social support was evaluated using a questionnaire consisting of 19 items comprising five dimensions of functional social support: material, affective, emotional, positive social interaction, and information [82]. For each item, the women indicated how often they experienced each type of available support: never, rarely, sometimes, often or always. The social support and social networks questionnaires have adequate psychometrics properties for the Brazilian population [83,84].

### Outcome

#### Self-rated health

The question used to measure SRH was: “Generally speaking, would you say that your health is: 1- excellent, 2- very good, 3- good, 4- fair, 5- poor”. This five-point scale was categorized as “good” (excellent, very good, good) and “poor” (fair, poor) SRH. The categorization of SRH adopted in this study was previously used [50].

Participants were divided into two groups according to the SRH at baseline and follow-up. Women who evaluated their health as good at both periods composed the Good SRH group, and Poor SRH group included women who evaluated their general health as poor at baseline and follow-up. Women who changed their SRH status between baseline and follow-up were separately analyzed because the main outcome of this study was consistent SRH. Changes in SRH during and after pregnancy are associated with many physical alterations and emotional problems [50]. Physical symptoms during pregnancy and shortly after delivery seem to be temporary for the majority of women. However, during the first months of motherhood, childcare is usually associated with tiredness, sleeping problems and low back pain [50], which are associated with poor SRH [60]. In this study, the groups of interest were women that, despite the physical and mental changes during pregnancy and postpartum period, had a consistent SRH.

### Covariates

The covariates were demographic and socioeconomic characteristics, health-related behaviours and health-reported diseases previous and during pregnancy. Demographic data were age, ethnicity and number of children in family after pregnancy, which were grouped into three categories. Age was categorized from 13 to 19, from 20 to 30, and 31 years old or more. Ethnicity, which was based on self-perception of skin colour, was categorized into White, Brown and Black; and number of children in family after pregnancy into 1 child, 2 or 3, and 4 or more children.

Socioeconomic characteristics were educational level, occupational context, family income, family structure and housing conditions. Educational level (from 0 to 8 years and 9 or more years of schooling), occupation (no paid work - women with no paid work, housewives or unemployed women; paid work – employed women with paid work), and family income (from 0 to 1 minimal wage and more than 1 minimal wage), were dichotomized. Family structure was evaluated by marital status and head of family; the family member responsible for the financial support of the household. Marital status was categorized as married living with partner, has a partner but not living with him and single without partner. Head of family was woman or husband/partner. Housing conditions was assessed by water plumbing supply (inside or outside the house), sewage (lack of sewage and pit sewage and general drainage), and number of people per room (1, 2 and 3 or more).

Social class was assessed at individual-level and then aggregated at neighbourhood-level. A standard classification of social class used in Brazil was employed. It is based on market power comprising a group of specific indicators and level of education of the head of household composed this variable. A set of points is assigned to these indicators and a final score defines the socioeconomic groups; A (highest), B, C, D, and E (lowest). Those with the highest scores represented the highest socioeconomic groups. In this study, the neighbourhoods were categorized as low, moderate and high socioeconomic status, based on the tertiles of the distribution of subjects into class B [85].

Health related behaviours assessed before pregnancy were smoking and alcohol consumption. In addition, the Brazilian version of T-ACE questionnaire was used to detect risky alcohol drinking before pregnancy. It is based on 5 items concerning self-perception of drinking habits. Two or more positive answers are indicative of increased chance of risky-drinking [86]. Smoking habits was assessed by the following question: “Did you smoke before pregnancy?”.

Self-reported diseases during pregnancy investigated were diabetes, hypertension and urinary infection.

### Ethical considerations

The study was approved by the Committee of Ethics and Research of the National School of Public Health - ENSP/FIOCRUZ (protocol CAAE nº 00779412.1.0000.52.40).

### Data management and statistical analysis

Multilevel logistic regression was used to test the association of neighbourhood and individual social capital with consistent SRH adjusted for neighbourhood and individual-level covariates. Consistency of SRH was a dichotomous outcome and logistic regression based on the *logit* function (logarithm of the odds) was performed with the predictive quasi likelihood second-order approximation procedures.

The multilevel structure comprised 685 individuals (level 1) grouped into 34 neighbourhoods (level 2). SPSS (Statistical Package for Social Sciences, version 17.0) was used in bivariate analyses. Continuous variables were compared using the *t* test and ANOVA, while categorical variables were analyzed using Chi-square test. Variables that presented *p* ≤ 0.20 in bivariate analysis were considered for the multivariate multilevel analysis.

The MLwiN 2.24 (Centre for Multilevel Modelling, University of Bristol, Bristol, UK) software was used for multilevel data analysis at two levels and all results are presented as Odds Ratios (ORs) with 95% Confidence Intervals (95% CIs). Four models were tested. The first model was composed by neighbourhood-level variables at 2nd level – neighbourhood social capital and social class. In the second model, individual social capital variables (social network and social support) were added. The third model included individual demographic and socioeconomic characteristics, and in the fourth model, the commonest self-reported disease during pregnancy (urinary infection) was added. Models 2, 3 and 4 were at 1st and 2nd level. Independent variables of each block were adjusted for each other, and those that remained significant at 5% (*p* ≤ 0.05) were retained in the analysis for adjustment in the next model to reduce discrepancy between the data and the model and reach an economic model with relatively few parameters [87]. The interaction term composed by neighbourhood social capital and individual social capital was added in the Model 1 to test a possible modifying effect of these variables on the outcome.

Separate bivariate analysis was conducted of the 222 women because their SRH status changed between baseline and six months after childbirth. Another reason for not considering them in the multilevel analysis was the low variability of SRH across the neighbourhoods (area level variable) when SRH was analyzed in four categories. The variance of SRH among neighbourhoods was tested through multinomial multilevel analysis and did not reach statistical significance. A pairwise analysis was performed between groups: Good SRH at baseline and follow-up; Poor SRH at baseline and follow-up; Poor SRH at baseline and Good at follow-up, Good SRH at baseline and Poor at follow-up. They were compared regarding socio-demographic characteristics, health-related behaviours and self-reported diseases, as well as individual social capital (social network domains and social support dimensions). Continuous variables were compared using the *t* test, while categorical variables were analyzed using Chi-square test.

## Results

The comparisons of demographic, socioeconomic and housing conditions characteristics between SRH groups are presented in Table 1. The study women were predominantly head of family (66.2%), without paid work (59.4%) and living in adequate housing conditions. Of the sample, 41.3% had schooling for more than 9 years, 85.7% had a family income more than 1 minimal wage and 70.8% were married. Most participants have 2 or 3 children, were Brown ethnicity and aged between 20 and 30 years (Table 1). Regarding socioeconomic covariates, the proportion of women who reported as head of family, living in poor housing conditions and with low schooling were statistically higher in the Poor SRH group (*p* ≤ 0.01). Bivariate analysis also showed the association between number of children in family after pregnancy and Brown and Black ethnicity with poor SRH (*p* ≤ 0.01).

**Table 1:** Socioeconomic and demographic characteristics in the self-rated health groups, State of Rio de Janeiro, Brazil, 2008-2009

N = 685		**Total (%)**	**Good SRH Baseline and Follow-up N = 597 (%)**	**Poor SRH Baseline and Follow-up N = 88 (%)**
Head of family	Woman	66.2	65.3	71.4
	Husband or partner	33.8	34.7	28.6
Occupational context	No paid work	59.4	57.6	64.8
	Paid work	40.6	42.4	35.2
People per room*	1	37.1	38.6	22.3
	2	34.9	34.9	44.4
	3 or more	28.0	26.5	33.3
Water supply***	Water plumbing supply inside house	81.8	84.1	69.3
	Water plumbing supply outside house	18.2	15.9	30.7
Sewage in your house	Lack of sewage or pit sewage	42.0	40.0	50.0
	General drainage	58.0	60.0	50.0
Schooling***	0-8 years	56.7	53.8	77.3
	9 or more years	43.3	46.2	22.7
Family income ^a^	0-1 Minimal wage	14.3	13.6	20.5
	More than 1 Minimal wage	85.7	86.4	79.5
Marital status	Married, living with partner	70.8	69.5	76.1
	Has a partner, not living with him	23.4	24.6	19.3
	Single without partner	5.8	5.9	4.6
Number of children in	1 child	36.3	40.5	15.9
family after pregnancy ***	2-3 children	45.7	44.9	53.4
	4 or more children	18.1	14.6	30.7
Ethnicity**	White	33.8	36.2	20.5
	Brown	42.5	41.4	47.7
	Black	23.7	22.4	31.8
Age	13 – 19	21.1	22.1	18.2
	20 – 30	57.8	57.8	52.3
	31+	21.1	20.1	29.5

** *p* ≤ .01, *** *p* ≤ .001.

*p *value refers to Chi-Square test.

^a^1 Brazilian Minimal Wage (BMW) = US$ 178 in 2008.

The comparison of health-related behaviours and self-reported diseases between SRH groups is presented in Table 2. Overall, the participants in both SRH groups reported that they consumed lower levels of alcohol and smoked less than before pregnancy. Whereas the prevalence of diabetes and hypertension before pregnancy was low (1.2% and 6.7%, respectively), urinary infection during pregnancy was common (45.5%). Self-reported diseases were higher in women with poor SRH (*p* ≤ 0.001).

**Table 2:** Health-related behaviours and self-reported diseases in the self-rated health groups, State of Rio de Janeiro, Brazil, 2008-2009

**N = 685**		**Total (%)**	**Good SRH Baseline and Follow-up N = 597 (%)**	**Poor SRH Baseline and Follow-up N = 88 (%)**
**Health-related behaviours**	Alcohol consumption			
	Do not drink alcohol	92.8	93.3	89.8
	No risk of alcoholism	5.3	4.7	6.8
	Risk of alcoholism	1.9	2.0	3.4
	Smoking before pregnancy			
	No	82.5	82.9	79.5
	Yes	17.5	17.1	20.5
**Self-reported diseases**	Diabetes***			
	No	98.8	99.3	94.3
	Yes	1.2	0.7	5.7
	Hypertension***			
	No	93.3	95.0	81.6
	Yes	6.7	5.0	18.4
	Urinary Infection***			
	No	54.5	59.1	42.0
	Yes	45.5	40.9	58.0

*** *p* ≤ .001.

*p* value refers to Chi-Square test.

There were statistically significant differences of individual social capital variables between the SRH groups. The mean scores of all social support dimensions were significantly higher in the Good SRH group compared to the Poor SRH group (*p* < 0.001). Women with poor SRH had less friendship social networks than those with good SRH. There was a borderline association between family social networks and Good SRH group (*p* = 0.063). Women in the Good SRH group reported significantly more participation in associations or social groups (*p* ≤ 0.05) (Table 3).

**Table 3:** Individual social capital scores (social support dimensions and social networks) in the self-rated health groups, State of Rio de Janeiro, Brazil, 2008-2009

**N = 685**		**Total**	**Good SRH Baseline and Follow-up N = 597**	**Poor SRH Baseline and Follow-up N = 88**
**Social Support dimensions, M (SD)**^**a**^	Affectionate support***	93.2 (13.6)	94.1 (12.6)	87.2 (17.9)
	Emotional support***	62.3 (20.1)	63.6 (18.9)	53.1 (25.4)
	Information support**	62.8 (19.3)	63.8 (18.3)	56.1 (23.7)
	Positive social interaction***	66.0 (19.0)	67.4 (17.4)	56.6 (23.6)
	Material support**	61.7 (20.0)	62.5 (19.6)	56.7 (22.5)
**Social network, (%)**^**b**^	Relatives			
	No relatives	18.4	16.1	24.1
	1 relative or more	81.6	83.9	75.9
	Friends***			
	No friends	40.8	36.6	55.2
	1 friend or more	59.2	63.4	44.8
	Member of any association or group*			
	No	70.7	68.0	78.4
	Yes	29.3	32.0	21.6

* *p* ≤ .05, ** *p* ≤ .01,*** *p* ≤ .001.

^a ^Mann–Whitney test.

^b ^Chi-Square test.

In the multivariate analysis four statistical models were developed to test the association of neighbourhood and individual social capital with SRH adjusted for covariates (Table 4). In Model 1, neighbourhood social capital and social class were adjusted for each other. Low neighbourhood social capital was statistically associated with poor SRH [OR 1.94 (95% CI: 1.02-3.88)]. Model 2 included individual social capital variables. Low neighbourhood social capital remained statistically associated with poor SRH [OR 2.10 (95% CI: 1.05-4.24)]. In addition, both individual social capital variables, social support (positive social interaction) [OR 0.78 (95% CI: 0.73-0.90)] and social networks (one or more friends) [OR 0.51 (95% CI: 0.32-0.80)] were inversely associated with poor SRH. In Model 3, neighbourhood-level variables were adjusted for individual socioeconomic and demographic covariates. Low neighbourhood social capital did not remain significantly associated with SRH. Apart from that, the statistical association between low social support and low social networks with poor SRH persisted. High social support was associated with 19% lower odds of poor SRH [OR 0.81 (95% CI: 0.73-0.90)] and social networks was associated with 40% lower odds of poor SRH [OR 0.60 (95% CI: 0.35-0.98)]. Of the socio-demographic covariates, only family income and age were not statistically associated with poor SRH.

**Table 4:** Adjusted odds ratio (OR) for poor self-rated health by the neighbourhood-level variables, individual social capital, socioeconomic and demographic variables and self-reported disease, State of Rio de Janeiro, Brazil, 2008-2009

**N = 685**	**Model 1**^**a **^**OR (95% CI)**	**Model 2**^**b **^**OR (95% CI)**	**Model 3**^**c**^**OR (95% CI)**	**Model 4**^**d **^**OR (95% CI)**
**Neighbourhood-level variables**				
**Neighbourhood Social Capital**				
High social capital (3rd tertil)	1	1	1	1
Moderate social capital (2nd tertil)	1.35 (0.74, 2.49)	1.42 (0.76, 2.65)	1.48 (0.74, 3.00)	1.47 (0.73, 3.01)
Low social capital (1st tertil)	1.94 (1.02, 3.88)*	2.10 (1.05, 4.24)**	1.97 (0.89, 4.39)	1.90 (0.85, 4.27)
**Social class**				
High% Social Class B	1	1	1	1
Moderate% Social Class B	0.71 (0.38, 1.34)	0.59 (0.31, 1.15)	0.62 (0.30, 1.32)	0.68 (0.32, 1.44)
Low% Social Class B	1.36 (0.76, 2.43)	1.16 (0.64, 2.11)	1.23 (0.62, 2.47)	1.39 (0.69, 2.84)
				
**Individual-level variables**				
**Individual Social Capital**				
Social support (per 10 units)		0.78 (0.73, 0.90)**	0.81(0.73, 0.90)***	0.82 (0.73, 0.90)***
Social network				
No friends		1	1	1
1 or more friends		0.51 (0.32, 0.80)**	0.60 (0.35, 0.98)*	0.61 (0.37, 0.99)*
**Sociodemographic**				
Water plumbing supply				
Inside the house			1	1
Outside the house			1.79 (1.02, 3.17)*	1.85 (1.05, 3.28)*
Family income				
≥ 2 Minimal wages			1	_
< 1 Minimal wage			1.12 (0.60, 2.14)	_
Number of children in family after pregnancy				
0-1 child			1	1
2 or 3 children			3.17 (1.54, 6.55)**	3.23 (1.66, 6.31)***
4 or more			3.15 (1.31, 7.57)**	3.39 (1.57, 7.31)***
Schooling				
≥ 9 years of schooling			1	1
0 to 8 years of schooling			2.01 (1.13, 3.61)**	2.06 (1.15, 3.71)**
Ethnicity				
White			1	1
Brown			1.95 (1.03, 3.70)*	2.02 (1.07, 3.85)**
Black			2.23 (1.12, 4.44)**	2.11 (1.19, 4.75)**
Age in years				
13-19			1	_
20-30			0.84 (0.40, 1.77)	_
31+			1.07 (0.46, 2.50)	_
**Self-reported disease**				
Urinary infection				2.11 (1.28, 3.49)**

*p ≤ 0.05. **p ≤ 0.01. ***p ≤ 0.001.

^a ^Contextual variables were adjusted for each other.

^b ^Contextual variables were adjusted for individual social capital (social networks and social support).

^c ^Contextual variables were adjusted for socioeconomic and demographic variables.

^d^ Contextual variables were adjusted for self-reported disease.

In the final adjusted model (Model 4), neighbourhood-level variables were adjusted for all individual variables, including self-reported urinary infection. Neighbourhood-level variables were not associated with SRH and the association between low social support and low social networks with poor SRH persisted in this final model. Women with higher levels of positive social interaction and those with one or more friends had 18% [OR 0.82 (95% CI: 0.73-0.90)] and 39% [OR 0.61 (95% CI: 0.37-0.99)] lower chances of reporting poor SRH. Among socio-demographic variables, water supply outside the house [OR 1.85 (95% CI: 1.05-3.28)] and low schooling [OR 2.06 (95% CI: 1.15-3.71)] were also associated with poor SRH. Women from Black and Brown ethnicity were 2.11 and 2.02 times more likely to report poor SRH compared to White women. Women with 2 or 3 children and those with 4 or more children were 3.23 and 3.39 times more likely to report poor SRH compared to those with no or one other child. Women reporting urinary infection had a higher likelihood of reporting poor SRH [OR 2.11 (95% CI: 1.28-3.49)].

In additional analysis that included the 222 women who changed SRH status, individual social capital was the major differential characteristic among the four groups. The mean score of affectionate and emotional supports (dimensions of social support) was higher among women with good SRH, and reduced significantly among those who changed from good SRH to poor SRH. Conversely, women who had poor SRH at baseline and changed to good SRH showed higher social support than those who remained with poor SRH at both assessments. In addition, in comparison to those with good SRH at baseline and poor SRH at follow-up, those with good SRH on both occasions had more relatives. However, no statistical differences were found concerning individual social capital between groups who changed their SRH status from good to poor, or from poor to good SRH. Data are presented in Additional file 3.

The interaction term, composed of neighbourhood social capital and individual social capital, was not statistically significant when added to the Model 1, and was therefore removed.

## Discussion

Despite the claim that neighbourhood social capital may be a contextual influence on the health of residents [88], and that there was a stronger associations between contextual social capital and health in women compared to men [89,90], this study found that neighbourhood social capital was more weakly associated with women’s SRH during pregnancy and up to 6 months after delivery than individual social capital and other socio-demographic characteristics. After adjustment for individual variables, no association was found between neighbourhood social capital and SRH. Therefore, findings of the present study do not support the hypothesis that neighbourhood social capital was related to degree of SRH during pregnancy and up to six months after childbirth.

Even though there is evidence of a moderate association between neighbourhood environment and health [10], some studies did not find an association between neighbourhood social capital and SRH [28,90]. Others found a substantial reduction in the association after adjusting for individual variables [31,91]. Findings from the Stockholm Public Health Cohort study revealed no or moderate associations between contextual social capital and SRH [28]; the presence or absence of such an association depended on how individual social capital was statistically analyzed. Similarly, the Caerphilly Health and Social Needs Survey found that the association between neighbourhood deprivation and SRH was substantially reduced after adjusting for individual socioeconomic status. In addition, the association was attenuated by perceptions of the neighbourhood and by housing problems [31]. In a study of 30 districts of Saskatchewan, Canada, the political and civic participation, proxies measures of social capital, were not related to SRH [92].

Our findings differ from other similar studies in Brazil that found an association between neighbourhood characteristics and SRH. In a cohort study involving public university workers, neighbourhood characteristics were associated with SRH after controlling for individual factors such as age, ethnicity, income, education, social class, home, and health conditions [24,93]. However, a large proportion of SRH variability was at the individual level and only 4% was explained by neighbourhood characteristics, which was reduced to 1% when individual variables were included in the model. Cremonese *et al.*[94] also reported that SRH depends more on individual characteristics than the socio-demographic context of neighbourhoods. According to Borges *et al.*[22], social capital may play an important role on SRH although the pattern of association differs according to the specific dimensions of social capital employed. They showed that social trust and civic participation as well as bridging social capital and social support remained associated with SRH in a group of adolescents after adjustment for other social capital indicators and confounders.

A positive association between social capital and SRH has been frequently reported [20,21,23,32,33]. In most studies, cross-sectional and ecological study designs were employed and demographic characteristics of the samples varied considerably. Variations involved demographic groups - adolescents, adults, elderly and pregnant women, and differences related to the levels of analysis - nations, states/regions, cities, neighbourhoods, and local institutions [33]. In addition, the interpretation of evidence is affected by the heterogeneous ways in which social capital was conceived and measured. Most studies used different composite indexes to characterize contextual social inequalities such as Index of Relative Socioeconomic Disadvantage (IRSD), neighbourhood socioeconomic status, Human Development Index (HDI), census-based composite indexes and Gini Index. Moreover, neighbourhood environment has been measured using the number and quality of local services and amenities [10], or by subjective evaluations obtained directly from local residents about their concerns and perceptions of the neighbourhood (about people and place), such as *proxy* measures or dimensions of neighbourhood social capital - social control, social trust, norms of reciprocity, feelings of safety (neighbourhood security), political efficacy and empowerment [15,16,44]. Therefore, valid comparisons between our findings and those from previous studies are difficult.

Although there was no association between neighbourhood social capital and poor SRH in the present study, there was a positive association between social support and social network and SRH. Therefore, the latter hypothesis was confirmed. The lower the social support and social networks, the higher the likelihood of maintaining poor SRH during pregnancy and 6 months postpartum. That finding accords with previous findings [56,60,95].

The period between pregnancy and 6 months after childbirth is a unique one in most women’s life. Individual social capital appears to be important for women, especially during pregnancy and after delivery. The lack of social support constitutes an important risk factor for maternal well-being during pregnancy [53]. Having low social support during pregnancy may reflect insufficient emotional support from the partner, family and/or friends that increase maternal stress, anxiety and depression, which in turn are related to well-being and SRH of pregnant women [50]. Psychosocial factors are also considered risk factors for undesirable pregnancy outcomes such as low birth weight, prematurity and intrauterine growth retardation [96,97]. Dissatisfaction with partner’s support increased the risk of poor SRH [48] and insufficient social support increased the risk of poor SRH in multiparas [60]. In the postpartum period, the lack of social support was the most consistent predictor of poor health outcomes [58]. In addition, social networks influenced health behaviours and lifestyle habits during pregnancy, including dietary habits and smoking [28,54,55,98]. Even though smoking during pregnancy was not assessed in the present study, previous studies showed that current smoking did not affect the association between social capital and SRH [31].

The strong influence of social support and social networks on women’s health [31,35] may be due to effective psychosocial resources, particularly social stability and social participation, that provide emotional and instrumental support [66]. During pregnancy and the months thereafter, the association between individual social capital and women’s SRH can be explained by the quality of personal and social resources [99]. As personal resources can be very limited compared to those from social ties, especially during pregnancy, social resources are necessary and should be accessible through strong social connections, which represents a sense of attachment or sharing one’s sentiments [99]. Moreover, high social participation was associated with lower odds of poor SRH in women after controlling confounders [100], probably because women have more ties with their neighbours than do men [101,102] and are more able to create and maintain local social networks that connect families and communities [103]. They are more likely to spend more time in the neighbourhood where they live, carrying out domestic tasks, going to supermarkets or grocery shops nearby or taking care of children and their elderly relatives [31], as well as participating in local volunteer work and/or religious groups [104].

Neighbourhood social capital may be affected by aspects of individual social capital that are associated with health [28]. Strong ties, such as social support and social networks, seem to mediate the association between neighbourhood social capital and consistent SRH [26]. However, in this study the interaction term was not statistically significant, suggesting that for pregnant women, the combined effect of neighbourhood social capital and individual social capital was not associated with poor SRH. Moreover, neighbourhood social capital was less important for those with consistent SRH during pregnancy and six months postpartum than individual social capital.

Women whose SRH status changed during the period of the study were not included in the multilevel analysis. By excluding them from the analysis, the study population may have been conditioned based on an effect of the exposure. However, the possibility of a ‘collider-stratification bias’ [105], a form of selection bias, was rejected by running a model without using postpartum SRH alone as the outcome and including all women regardless of SRH during pregnancy (data not shown). The results were very similar, suggesting that selection bias did not occur.

Additional analysis showed that individual social capital differed between groups whose SRH status changed. In addition, the possible influence of individual social capital in women not changing from good to poor levels of SRH over a period of great stress and transition was not confirmed. Conversely, individual social capital was associated with consistent SRH. The findings indicate that it is unlikely that including women who changed their SRH status over time in the overall analysis would affect the results. The influence of individual and neighbourhood social capital on SRH in women whose pregnancies were aborted or miscarried should be considered in future studies.

The findings of this study have several important implications, but are not without limitations. Recruiting pregnant women from public antenatal care units might affect the generalizability of our findings resulting in a homogeneous sample concerning some socioeconomic characteristics. This may explain the lack of association between social class and SRH. The number of women per neighbourhood was slightly below the ideal and might have affected the power of the study [72]. Our study is limited to pregnant and postpartum women, and considering the specificity of the sample employed in the present study, our findings must be viewed with caution and must not be generalized.

A point to be taken into account when considering our findings is that urinary infection was prevalent in our sample (45.5%), and was statistically higher in women with poor SRH. The prevalence of urinary infection was slightly higher than in other studies [62,106]. The presence of urinary infection was based on self-reports and subject to misreporting. The reported associations in the final model did not change significantly when the variable urinary infection was removed. Future studies should consider collecting data about complications such as ‘morning sickness’ in early pregnancy or backache, perineal problems during pregnancy and postpartum, and complications of caesarean section postnatally, to validate subjective measures of health.

Positive features of the study were the use of social support and social networks questionnaires with adequate psychometric properties for the Brazilian population. The neighbourhood social capital questionnaire had been used in Brazilian populations and had good reliability. The data collection was standardized and performed by trained interviewers reducing information bias. In addition, the response rate was high and losses to follow-up were low.

There are some points related to the social capital assessment that should to be considered in future studies. One is the measure *per si*. There is no consensus on how to measure social capital. Contextual social features have been routinely assessed using aggregated measures and indexes, like percentage of poverty or median of income, mainly those available in census databases [10]. In this study, neighbourhood social capital was assessed individually and then aggregated at area level. However, the characterization of places and neighbourhoods by interviewers was not done. Social epidemiologists need to assess specific characteristics of places and neighbourhoods through primary data collection (compositional characteristics), paying attention to resources embedded in social networks [26].

The present study enhances knowledge on social capital and health in several ways. Our findings indicate a relationship between individual social capital and SRH. In addition, multilevel analysis is an adequate statistical approach to evaluate simultaneously the role of neighbourhood-level and individual factors on health. Above all, more attention was given to low socioeconomic status women, where undesirable pregnancy outcomes rates remain relatively high in other low and middle income countries. Knowledge about how proximal and distal social determinants affect women’s health during pregnancy and the initial period of motherhood has important policy implications on the health and social areas. The identification on what level of social capital was more important to womenÂ´s health is relevant in developing interventions. Although social inequalities are viewed as a broad and unspecific determinants of health, our findings suggest that there are specific social risk factors during and after pregnancy that should be taken into account to improve both maternal and child health.

## Conclusions

Low individual social capital during pregnancy, such as social support and social networks, independently influenced women who had consistently poor SRH whereas neighbourhood social capital did not affect their SRH during pregnancy and the postpartum period. From pregnancy and up to six months after delivery the effect of individual social capital better explained consistent SRH in women than did neighbourhood social capital.

## Competing interests

The authors declare that they have no competing interests.

## Authors’ contributions

GL was involved in design of the study, acquisition of data, analysis and interpretation of the data, interpretation of the results and drafted the manuscript. M do CL helped design the study, interpreted the data and reviewed the manuscript. AS was involved in interpretation of the data and revising the manuscript. MV was involved in the conception and design of the study, developed the statistical framework for data analysis, and helped to draft the manuscript. All authors read and approved the final manuscript.

## Pre-publication history

The pre-publication history for this paper can be accessed here:

http://www.biomedcentral.com/1471-2393/13/1/prepub

## Supplementary Material

Additional file 1 Social capital questionnaire. Click here for file

Additional file 2 Social networks questionnaire. Click here for file

Additional file 3 Socioeconomic and demographic characteristics, health-related behaviours, and individual social capital scores in all groups of SRH. Click here for file
