# Supplementary material for: The association of neighbourhood and individual social capital with consistent self-rated health: a longitudinal study in Brazilian pregnant and postpartum women
Source: BMC Pregnancy Childbirth. 2013 Jan 16;13:1. doi: 10.1186/1471-2393-13-1 (PMC3556498; doi:10.1186/1471-2393-13-1)
Supplement: Additional file 1 — Social capital questionnaire. [file 1471-2393-13-1-S1.docx]

ADDITIONAL FILE 1

| **Social Capital Questionnaire** |
| --- |
| **Social Trust answers:**  **How strongly would you agree with the following statements?**  People around here are willing to help their neighbors/ This is a close-knit neighbourhood / People in this neighbourhood can be trusted/ People in this neighbourhood generally do not get along with each other / People in this neighbourhood do not share the same values  *(0) Disagree; (1) Neither agree nor disagree; (2) Agree.*  **How comfortable would you be asking a neighbor in each of the following situations?**  To collect a prescription from the chemist for you if you were in bed / If you could talk to them about a personal problem that you had / To look after your child if you needed to go out for a while / To lend you R$ 15 for a couple of days  *(0)Would not ask neighbor; (1) Very uncomfortable; (2) Fairly uncomfortable; (3) Fairly comfortable; (4) Very comfortable* |
| **Social Control answers:**  **How strongly would you agree with the following statements?**  I can count on my neighbors to intervene if children were skipping or missing school and hanging out on a street corner / I can count on my neighbors to intervene if children were spray-painting graffiti on a local building / I can count on my neighbors to intervene if children were showing disrespect to an adult / I can count on my neighbors to intervene if a fight broke out in front of our house / I can count on my neighbors to intervene if the health center closest to my house was threatened with budget cuts  *(0) Disagree; (1) Neither agree nor disagree; (2) Agree.* |
| ***Political Efficacy answers:***  **How strongly would you agree with the following statements?**  I don't think public officials care much about what people like me think / Political parties are only interested in people's votes, but not their opinions / Generally speaking, people we elect to congress lose touch with the people pretty quickly /People like me have no say in what the government does.   1. *Disagree; (1) Neither agree nor disagree; (2) Agree.*   **Neighbourhood Security answers:**  **Now let's talk about violence in your local area. When was the last time that one of the following have occurred in your neighbourhood:**  A fight in which a weapon was used / A violent argument between neighbors / A gang fight / A sexual assault or rape / A robbery or mugging / A drug-related episode / A homicide.  *(0) Have never occurred; (1) More than 12 months ago; (2) In the past 12 months; (3) In the past 6 months; (4) In the past 3 months.*  **While you have lived in this neighbourhood, has anyone ever used violence, such as mugging, fight, assault or other, against you or any member of your household?**  *( ) Yes ( ) No* |
