# Supplementary material for: The association of neighbourhood and individual social capital with consistent self-rated health: a longitudinal study in Brazilian pregnant and postpartum women
Source: BMC Pregnancy Childbirth. 2013 Jan 16;13:1. doi: 10.1186/1471-2393-13-1 (PMC3556498; doi:10.1186/1471-2393-13-1)
Supplement: Additional file 2 — Social networks questionnaire. [file 1471-2393-13-1-S2.docx]

ADDITIONAL FILE 2

| **Social Networks Questionnaire** |
| --- |
| **Are you now or have been (last 3 years) member of any association?** *( ) Yes ( ) No*  **Which association(s): Tick as many as you like**  Religious group *( ) Yes ( ) No*  Community association *( ) Yes ( ) No*  Parent-Teacher Association *( ) Yes ( ) No*  Sport association *( ) Yes ( ) No*  Union *( ) Yes ( ) No*  Political party *( ) Yes ( ) No*  Other _______________  **How often do you attend religious services? Tick only one**  (5) Almost daily; (4) About once a week; (3) About once a month; (2) Once every few months; (1) Almost never (0) Never.  **How often do you watch news programmes on TV? Tick only one**  (5) Almost daily; (4) About once a week; (3) About once a month; (2) Once every few months; (1) Almost never (0) Never.  **How often do you read daily newspapers? Tick only one**  (5) Almost daily; (4) About once a week; (3) About once a month; (2) Once every few months; (1) Almost never (0) Never.  **Is there any relative with whom you feel comfortable and can talk about almost everything?**  *( ) Yes ( ) No*  **Is there a friend with whom you feel comfortable and can talk about almost everything?**  *( ) Yes ( ) No* |
