# Supplementary material for: The association of neighbourhood and individual social capital with consistent self-rated health: a longitudinal study in Brazilian pregnant and postpartum women
Source: BMC Pregnancy Childbirth. 2013 Jan 16;13:1. doi: 10.1186/1471-2393-13-1 (PMC3556498; doi:10.1186/1471-2393-13-1)
Supplement: Additional file 3 — Socioeconomic and demographic characteristics, health-related behaviours, and individual social capital scores in all groups of SRH. [file 1471-2393-13-1-S3.doc]

ADDITIONAL FILE 3

| **N = 907** | **Total (%)** | **Good SRH Baseline**  **and**  **Follow-up**  **N=597 (%)^a^** | **Poor SRH Baseline and Follow-up**  **N=88 (%)^b^** | **Poor SRH**  **at Baseline/**  **Good at Follow-up**  **N=128 (%)^c^** | **Good SRH at Baseline/ Poor at Follow-up**  **N=94 (%)^d^** | | **Pairwise analysis** |
| --- | --- | --- | --- | --- | --- | --- | --- |
| **Head of family** |  |  |  |  |  | |  |
| Woman | 66.2 | 65.3 | 71.4 | 65.2 | 67.9 | |  |
| Husband or partner | 33.8 | 34.7 | 28.6 | 34.8 | 32.1 | |  |
| **Occupational context** |  |  |  |  |  | |  |
| No paid work | 59.4 | 57.6 | 64.8 | 64.1 | 59.6 | |  |
| Paid work | 40.6 | 42.4 | 35.2 | 35.9 | 40.4 | |  |
| **People per room*** |  |  |  |  |  | | *a-b*; b-d** |
| 1 | 37.1 | 38.7 | 22.2 | 36.1 | 44.9 | |  |
| 2 | 34.9 | 34.9 | 44.4 | 27.9 | 28.6 | |  |
| 3 or more | 28.0 | 26.5 | 33.3 | 36.1 | 26.5 | |  |
| **Water plumbing supply***** |  |  |  |  |  | | *a-b*; b-d** |
| Inside house | 81.8 | 84.1 | 69.3 | 79.7 | 81.9 | |  |
| Outside house | 18.2 | 15.9 | 30.7 | 20.3 | 18.1 | |  |
| **Sewage in your house** |  |  |  |  |  | |  |
| Lack of sewage or pit sewage | 42.0 | 40.0 | 50.0 | 48.4 | 38.3 | |  |
| General drainage | 58.0 | 60.0 | 50.0 | 51.6 | 61.7 | |  |
| **Schooling***** |  |  |  |  |  | | *a-b** |
| 0-8 year | 58.7 | 53.8 | 77.3 | 68.0 | 59.6 | |  |
| More than 9 year | 41.3 | 46.2 | 22.7 | 32.0 | 40.4 | |  |
| **Family income** |  |  |  |  |  | |  |
| 0-1 Minimal wage | 14.3 | 13.6 | 20.5 | 13.3 | 14.9 | |  |
| More than 1 Minimal wage | 85.7 | 86.4 | 79. 5 | 86.7 | 85.1 | |  |
| **Marital status** |  |  |  |  |  | |  |
| Married, living with partner | 70.8 | 69.5 | 76.1 | 73.4 | 70.2 | |  |
| Has a partner, not living with him | 23.4 | 24.6 | 19.3 | 21.1 | 22.3 | |  |
| Single without partner | 5.8 | 5.9 | 4.5 | 5.5 | 7.4 | |  |
| **Number of children in family after delivery***** |  |  |  |  | *a-b*; a-d*; a-d*; b-c** | | |
| 1 child | 36.2 | 40.5 | 15.9 | 34.4 | 29.8 |  | |
| 2-3 children | 45.7 | 44.9 | 53.4 | 45.6 | 43.6 |  | |
| 4 or more children | 18.1 | 14.6 | 30.7 | 20.0 | 26.6 |  | |
| **Ethnicity**** |  |  |  |  |  | *a-b*; b-d** | |
| White | 33.8 | 36.2 | 20.5 | 29.7 | 36.6 |  | |
| Brown | 42.5 | 41.4 | 47.7 | 50.0 | 34.4 |  | |
| Black | 23.7 | 22.4 | 31.8 | 20.3 | 29.0 |  | |
| **Age** |  |  |  |  |  | *b-d** | |
| 13 – 19 | 21.1 | 22.1 | 18.2 | 19.5 | 19.1 |  | |
| 20 – 30 | 57.9 | 57.8 | 52.3 | 56.3 | 66.0 |  | |
| 31+ | 21.1 | 20.1 | 29.5 | 24.2 | 14.9 |  | |

**Table 5: Socioeconomic and demographic characteristics in all groups of SRH, State of Rio de Janeiro, Brazil, 2008-2009**

***p*≤ .01, *** *p* ≤ .001 *p* value refers to Chi-Square test

**Differences between groups** - *^a^ Good SRH at Baseline and follow-up; ^b^ Poor SRH at Baseline and follow-up; ^c^ Poor SRH at Baseline and Good at follow-up; ^d^ Good SRH at Baseline and Poor at follow-up*

*a-b*; a-c*; a-d*; b-c*; b-d*; c-d* - differences between groups at significant level (p* ≤ .05)

**Table 6: Health-related behaviours and self-reported diseases in all groups of SRH, State of Rio de Janeiro, Brazil, 2008-2009**

| **N = 907** | **Total**  **(%)** | **Good SRH Baseline and Follow-up**  **N=597 (%)^a^** | **Poor SRH Baseline and Follow-up**  **N=88 (%)^b^** | **Poor SRH**  **at Baseline/**  **Good at Follow-up**  **N=128 (%)^c^** | **Good SRH at Baseline/ Poor at Follow-up**  **N=94 (%)^d^** | **Pairwise analysis** |
| --- | --- | --- | --- | --- | --- | --- |
| **Health-related behaviors** |  |  |  |  |  |  |
| Alcohol consumption |  |  |  |  |  |  |
| Do not drink alcohol | 92.8 | 93.3 | 89.8 | 93.0 | 92.6 |  |
| No risk of alcoholism | 5.3 | 4.7 | 6.8 | 5.5 | 7.4 |  |
| Risk of alcoholism | 1.9 | 2.0 | 3.4 | 1.6 | 0 |  |
| Smoking before pregnancy |  |  |  |  |  |  |
| No | 82.5 | 82.9 | 79.5 | 76.6 | 79.8 |  |
| Yes | 17.5 | 17.1 | 20.5 | 23.4 | 20.2 |  |
| **Self-reported diseases** |  |  |  |  |  |  |
| Diabetes*** |  |  |  |  |  | *a-b*; b-c** |
| No | 98.8 | 99.3 | 94.3 | 99.2 | 98.9 |  |
| Yes | 1.2 | 0.7 | 5.7 | 0.8 | 1.1 |  |
| Hypertension*** |  |  |  |  | *a-b*; a-c*;a-d** | |
| No | 93.3 | 95.0 | 81.6 | 86.5 | 85.1 |  |
| Yes | 6.7 | 5.0 | 18.4 | 13.5 | 14.9 |  |
| Urinary Infection*** |  |  |  |  | *a-b*; a-c*; a-d** | |
| No | 54.5 | 59.1 | 42.0 | 48.0 | 45.2 |  |
| Yes | 45.5 | 40.9 | 58.0 | 52.0 | 54.8 |  |
|  |  |  |  |  |  |  |

*** *p* ≤ .001 *p* value refers to Chi-Square test

**Differences between groups** - *^a^ Good SRH at Baseline and follow-up; ^b^ Poor SRH at Baseline and follow-up; ^c^ Poor SRH at Baseline and Good at follow-up; ^d^ Good SRH at Baseline and Poor at follow-up*

*a-b*; a-c*; a-d*; b-c*; b-d*; c-d* - differences between groups at significant level (p* ≤ .05)

**Table 7: Individual social capital scores (social support dimensions and social network domains) in all SRH groups, State of Rio de Janeiro, Brazil, 2008-2009**

| **N = 907** | **Total**  **(%)** | **Good SRH Baseline and Follow-up**  **N=597 (%)^a^** | **Poor SRH Baseline and Follow-up**  **N=88 (%)^b^** | **Poor SRH**  **at Baseline/**  **Good at Follow-up**  **N=128 (%)^c^** | **Good SRH at Baseline/ Poor at Follow-up**  **N=94 (%)^d^** | **Pairwise analysis** |
| --- | --- | --- | --- | --- | --- | --- |
| **Social Support**  **dimensions, M (SD)^1^** |  |  |  |  |  |  |
| Affectionate support*** | 93.2 (13.6) | 94.1 (12.6) | 87.2 (17.9) | 90.7 (17.4) | 92.7 (12.7) | *a-b* a-c*;*  *b-c*; b-d** |
| Emotional support*** | 62.3 (20.1) | 63.6 (18.9) | 53.1 (25.4) | 60.0 (21.7) | 58.4 (21.9) | *a-b*; a-d*;*  *b-c** |
| Information support** | 62.8 (19.3) | 63.8 (18.3) | 56.1 (23.7) | 60.0 (21.2) | 58.6 (24.1) | *a-b** |
| Positive social interaction*** | 66.0 (19.0) | 67.4 (17.4) | 56.6 (23.6) | 60.6 (21.2) | 63.2 (18.8) | *a-b*; a-c*;*  *a-d** |
| Material support** | 61.7 (20.0) | 62.5 (19.6) | 56.7 (22.5) | 56.3 (21.4) | 56.0 (20.81 | *a-b*; a-c*;*  *a-d** |
| **Social Networks, (%)^2^** |  |  |  |  |  |  |
| Relatives * |  |  |  |  |  | *a-d** |
| No relatives | 18.4 | 16.1 | 24.1 | 19.2 | 26.6 |  |
| 1 relative or more | 81.6 | 83.9 | 75.9 | 80.8 | 73.4 |  |
| Friends*** |  |  |  |  |  | *a-b*; a-c** |
| No friends | 40.8 | 36.6 | 55.2 | 49.6 | 45.7 |  |
| 1 friend or more | 59.2 | 63.4 | 44.8 | 50.4 | 54.3 |  |
| Member of any association or group | |  |  |  |  | *a-b** |
| No | 70.7 | 68.0 | 78.4 | 75.0 | 74.5 |  |
| Yes | 29.3 | 32.0 | 21.6 | 25.0 | 25.5 |  |
|  |  |  |  |  |  |  |

* *p* ≤ .05, ** *p* ≤ .01,*** *p* ≤ .001 *p* value refers to ^1^ Mann-Whitney test^; 2^ Chi-Square test

**Differences between groups** - *^a^ Good SRH at Baseline and follow-up; ^b^ Poor SRH at Baseline and follow-up; ^c^Poor SRH at Baseline and Good at follow-up; ^d^ Good SRH at Baseline and Poor at follow-up*

*a-b*; a-c*; a-d*; b-c*; b-d*; c-d* - differences between groups at significant level (p* ≤ .05)
